# 肺NUT癌1例报告及文献复习

**DOI:** 10.3779/j.issn.1009-3419.2020.102.44

**Published:** 2021-01-20

**Authors:** 小琴 刘, 艳莹 李, 敏 余, 麟 周

**Affiliations:** 1 610400 成都，金堂县第一人民医院肿瘤科 Department of Oncology, First People's Hospital of Jintang County, Chengdu 610400, China; 2 610041 成都，四川大学华西医院胸部肿瘤科 Department of Thoracic Oncology, West China Hospital, Sichuan University, Chengdu 610041, China

**Keywords:** 肺肿瘤, 放疗, 化疗, 生存期, Lung neoplasms, Radiotherapy, Chemotherapy, Overall survival

## Abstract

NUT癌是以染色体15q14上睾丸核蛋白（nuclear protein in testis, NUTM1）重排为特征的一种罕见的恶性程度极高的肿瘤，其发病机制及治疗方法目前尚不明确，其预后极差。因其好发部位主要集中在躯体中线位置，如眼眶、鼻腔、上颚、纵隔等，故又称为中线癌。四川大学华西医院胸部肿瘤科收治了1例肺NUT癌患者，为70岁老年男性，初诊为体检发现左肺门占位，术后病理提示低分化癌，经荧光标记的原位杂交技术证实为NUT癌，术后经化疗、抗血管治疗、放疗等综合治疗获得了较长的生存期。本文结合文献回顾报告了肺NUT癌这一少见实体瘤的临床、病理特征及治疗策略。

## Abstract (en)

NUT carcinoma is a rare, high lethal cancer which feature as the rearrangement of the nuclear protein in testis (NUT) gene on chromosome 15q14, and its pathogenesis and treatment is not yet clear, the prognosis is poor. Due to NUT carcinoma often occurred on the middle position of the body, such as eyes, nose and palate, mediastinum, so it is also called NUT midline carcinoma (NMC). In this case, we report a 70 years old man who was diagnosed as primary pulmonary NUT carcinoma in the Department of Thoracic Oncology, West China Hospital of Sichuan University. A tumor was found at the left hilus pulmonis when his physical examination without any symptom. Postoperative pathological showed poorly differentiated carcinoma, confirmed by fluorescence *in situ* hybridization technique for the NUT carcinoma. After operation, chemotherapy, antiangiogenesis therapy, and radiotherapy were given, and he got a long lifetime. Combined with the literature review, we report the clinical and pathological features, and treatment strategies of the rare pulmonary NUT carcinoma case.

NUT癌（NUT carcinoma）是一种组织起源不明的罕见的高度侵袭性恶性肿瘤，伴有睾丸核蛋白（nuclear protein in testis, NUT）基因重排（位于15号染色体）^[1]^。最初被认为好发于儿童和青少年，但后来病例数报道的增多提示NUT癌可发生于任何年龄的患者，男女发病率无明显差异^[2-4]^。因其好发部位在人体中线器官，如头面部、肺、纵隔等部位，因此又被称为中线癌（NUT middle carcinoma, NMC）^[5, 6]^。据目前最新报道^[7]^，NMC的中位生存期仅5个月。肺NUT癌的病例报道不多，且预后更差，Sholl等^[8]^回顾性报道了8例原发性肺NUT癌，分析了其病理、临床、影像方面的特征，为早期识别该类肿瘤提供了宝贵的意见。此外，还有一些肺NUT癌的个案报道，均提示肺NUT癌的诊疗存在巨大困难^[9-11]^。本文报道1例肺NUT癌经过手术、化疗、放疗、抗血管治疗等综合治疗后获得了较长的生存期。

## 1 病例介绍

患者为1例70岁老年男性，因体检发现左肺上叶占位就诊，就诊时患者无咳嗽、咳痰、咯血、胸痛等不适，既往无吸烟饮酒史，家族史无特殊。正电子发射计算机断层显像（positron emission tomography-computed tomography, PET-CT）提示：左肺上叶恶性占位伴左肺门淋巴结转移；2018年6月15日于当地医院行胸腔镜并中转开胸行左肺上叶切除术+区域淋巴结清扫术。术后病理示：（左肺上叶）神经内分泌癌伴腺癌。后于四川大学华西医院病理会诊，（H1808692 2018年7月25日）示：（左肺上叶）非小细胞肺癌（non-small cell lung cancer, NSCLC），为低分化癌。免疫组化示：肿瘤细胞PCK（部分+）、P63（+）、P40（-）、CK5/6（部分+）、CD56（+）、Syna（-）、CgA（-）、CK7（-）、Napsin（-）、TTF-1（-）、Ki-67/MIB-1（+, 30%）；结合形态学及免疫组化，倾向NUT癌。基因检测示：未检测到间变性淋巴瘤激酶（anaplastic lymphoma kinase, *ALK*）、表皮生长因子受体（epidermal growth factor receptor, *EGFR*）、*KRAS*、*BRAF*、*ERBB2*、*ROS1*、*NRAS*等基因突变。行NUT/荧光原位杂交技术（fluorescence *in situ* hybridization, FISH）检测：检出*NUT*基因易位。术后病理诊断：左肺上叶NUT癌伴肺门淋巴结转移[pT3N1M0 IIIa期，*ALK*（-）、*ROS1*（-）]。

随后患者接受了3个周期EP（依托泊苷+顺铂）联合贝伐珠单抗治疗，因患者心功能欠佳更改化疗方案为EC（依托泊苷+卡铂）+贝伐珠单抗，化疗1个周期后定期随访。辅助化疗完成后10个月行胸部CT发现左肺门区、降主动脉旁见增多软组织密度影，提示局部肿瘤复发可能（图 1A）。随即接受胸部复发病灶放疗，调强适形放疗（intensity modulated radiation therapy, IMRT）40 Gy/20 F/4 wk（图 1C、图 1D）。放疗完成后复查胸部CT提示肿瘤消退明显（图 1B）。遂行单药E（依托泊苷）化疗，1个周期后因不能耐受化疗不良反应而终止化疗。胸部放疗结束后2个月复查CT提示左侧膈肌肿物，考虑转移可能性大（图 2A），随即行左侧膈肌肿物放疗，IMRT 40 Gy/20 F/4 wk（图 2C、图 2D）。膈肌放疗过程中出现左侧臀部及左侧大腿放射性疼痛，疼痛数字评分法（numerical rating scale, NRS）评分6分-8分。行骨盆CT检查，提示左侧骶髂关节处软组织影，且局部骨质破坏严重（图 3A），再次安排左侧骶髂关节放疗，IMRT 40 Gy/10 F/2 wk（图 3C、图 3D）。放疗结束后左侧臀部疼痛较前明显缓解。左侧骶髂关节完成放疗后1个月复查CT提示：左侧膈肌及左侧骶髂关节肿瘤较前退缩明显（图 2B、图 3B），但双侧颈部、锁骨上、上纵隔淋巴结多发增大，右肺结节，右侧肋骨骨质破坏，考虑肿瘤进展（图 4A、图 4B、图 5A、图 6A、图 6B）。患者一般情况欠佳，拟行低剂量放疗联合免疫治疗，完善程序性死亡受体配体1（programmed cell death ligand 1, PD-L1）检测，免疫组化：PD-L1（22C3）（-）。遂行双侧锁骨上淋巴结、纵隔淋巴结低剂量放疗，IMRT 6 Gy/3 F/3 d（图 4E），同时右下肺内转移灶放疗，SBRT 30 Gy/3 F/7 d（图 5C、图 5D），未行肋骨转移灶放疗，放疗总结详见表 1。放疗完成后予以信迪利单抗200 mg，*ivgtt*，*q3w*，免疫治疗，共2个周期。放疗结束后2个月复查CT提示：颈部及锁骨上、上纵隔淋巴结较前无明显变化，肺内结节明显增大，肋骨病灶较前明显增大，疗效评估疾病进展（progressive disease, PD）（图 4C、图 4D、图 5B、图 6C、图 6D）。考虑患者一般情况欠佳，体能状态（performance status, PS）评分2分-3分，建议口服安罗替尼，目前口服安罗替尼中。

**图 1:**
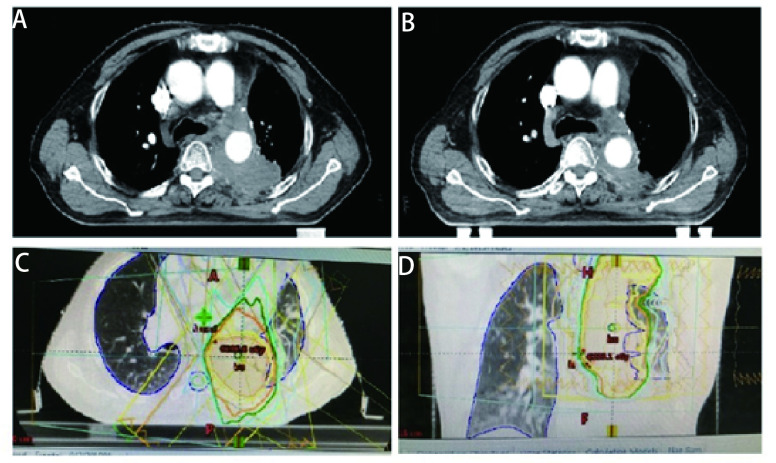
首次左侧纵隔内复发及放疗后。A：左侧纵隔内复发；B：经40 Gy/20 F放疗后肿瘤明显退缩；C：左侧纵隔肿物放疗靶区图（冠状位）；D：左侧纵隔肿物放疗靶区图（矢状位）。 First recurrence at the left side of the mediastinum and after radiotherapy. A: recurrence at the left side of the mediastinum; B: Tumor significantly shrink after radiotherapy with 40 Gy/20 F; C: Target volume of the mediastinum tumor (coronal); D: Target volume of the mediastinum tumor (sagittal).

**图 2:**
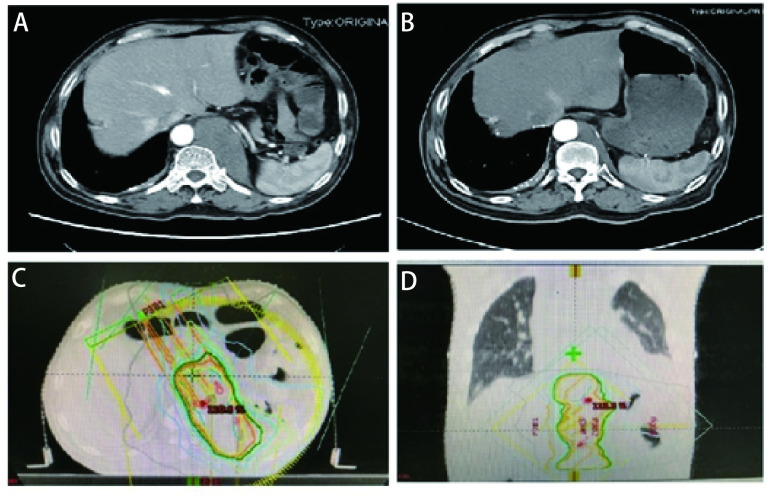
左侧膈肌转移灶经放疗后肿瘤明显退缩。A：左侧膈肌转移；B：经40 Gy/20 F放疗后肿瘤明显退缩；C：左侧膈肿物放疗靶区图（冠状位）；D：左侧膈肿物放疗靶区图（矢状位）。 Metastasis at the left side of the diaphragm, and tumor significantly shrink after radiotherapy. A: Metastasis at the left side of the diaphragm; B: Tumor significantly shrink after radiotherapy with 40 Gy/20 F; C: Target volume of the diaphragm tumor (coronal); D: Target volume of the diaphragm tumor (sagittal).

**图 3:**
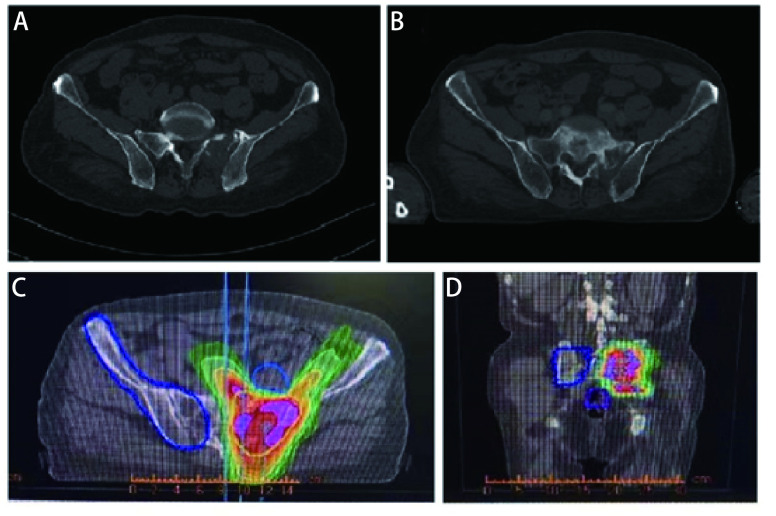
左侧骶髂关节转移灶经放疗后肿瘤明显退缩。A：左侧骶髂关节软组织影及骨质破坏；B：经40 Gy/20 F放疗后肿瘤明显退缩；C：左侧骶髂关节转移灶放疗靶区图（冠状位）；D：左侧骶髂关节转移灶放疗靶区图（矢状位）。 Metastasis at the left side of the sacroiliac joint, and tumor significantly shrink after radiotherapy. A: Metastasis at the left side of the sacroiliac joint; B: Tumor significantly shrink after radiotherapy with 40 Gy/20F; C: Target volume of the diaphragm tumor (coronal); D: Target volume of the diaphragm tumor (sagittal).

**图 4:**
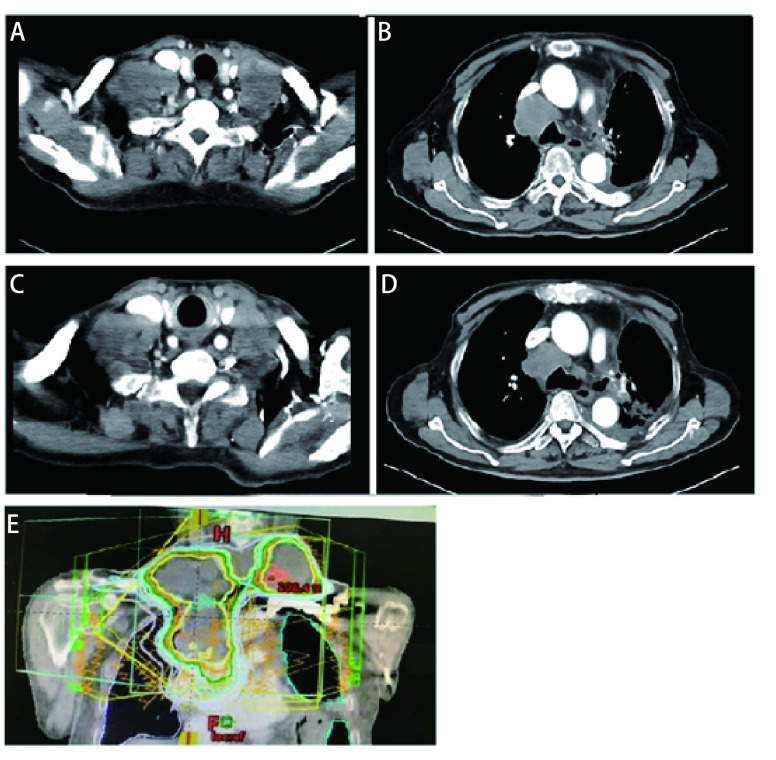
锁骨上及纵隔淋巴结增大，经低剂量放疗后肿瘤无明显变化。A：双侧锁骨上淋巴结增大；B：纵隔淋巴结增大；C、D：经低剂量6 Gy/3 F放疗后肿瘤无明显变化；E：锁骨上及纵隔转移灶放疗靶区图（矢状位）。 Metastasis at the supraclavicular and mediastinal lymph nodes, and no significant change after low dose radiotherapy. A: Metastasis at the bilateral supraclavicular lymph nodes; B: Metastasis at the mediastinal lymph nodes; C, D: Tumor have no significant change after low dose radiotherapy with 6 Gy/3 F; E: Target volume of the supraclavicular and mediastinal lymph nodes (sagittal).

**图 5:**
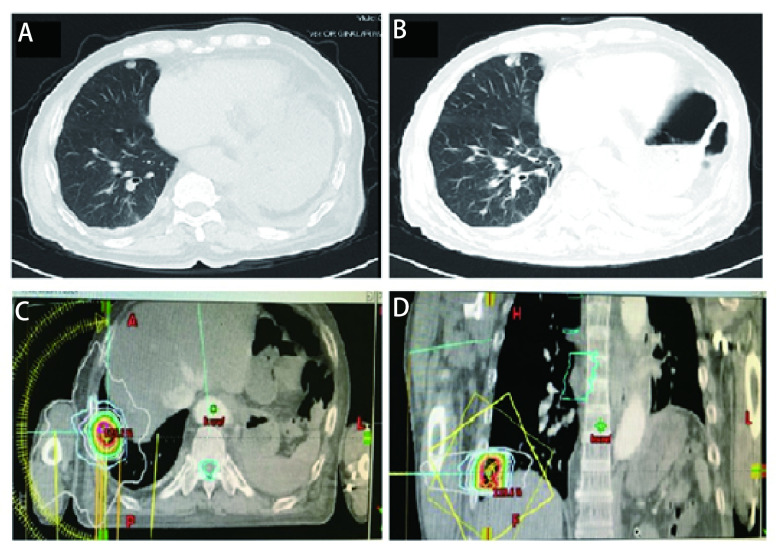
右肺下叶转移灶经立体定向放疗后肿瘤较前进展。A：右肺下叶新发转移灶；B：经30 Gy/3 F放疗后肿瘤较前增大；C：右肺下叶转移灶放疗靶区图（冠状位）；D：右肺下叶转移灶放疗靶区图（矢状位）。 Metastasis at the inferior lobe of right lung, and tumor progression after SBRT. A: Metastasis at inferior lobe of right lung; B: Tumor progressed after SBRT radiotherapy with 30 Gy/3 F; C: Target volume of the tumor at the inferior lobe of right lung (coronal); D: Target volume of the tumor at the inferior lobe of right lung (sagittal). SBRT: stereotactic body radiation therapy.

**图 6:**
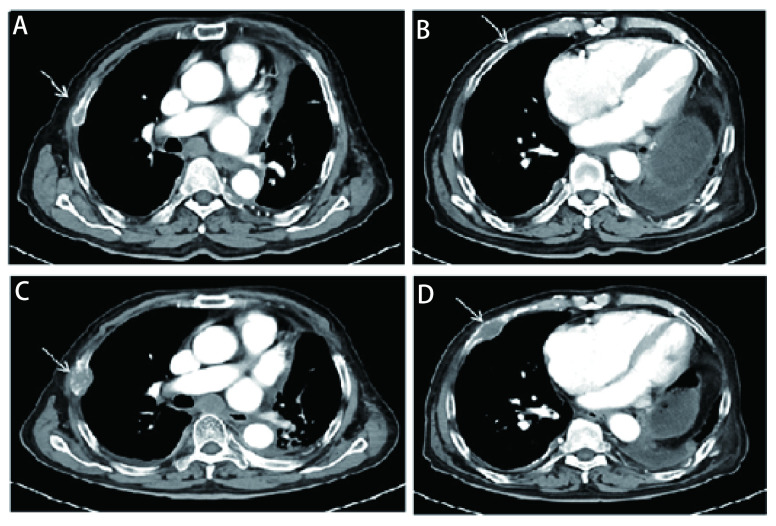
肋骨未经放疗快速进展。A、B：箭头处可见微小骨质破坏；C、D：箭头处可见骨质破坏加重，肿瘤进展。 Metastasis at the ribs, and tumor rapidly progressed without radiotherapy. A, B: Arrow marked the little broken of the ribs; C, D: Arrow marked the broken of the ribs enlarged.

**表 1:** 本病例放疗情况 Radiotherapy in this case

Site	Dosage	Technique	Evaluation
Hilum of left lung	40 Gy/20 F/4 wk	IMRT	PR
Left diaphragmatic	40 Gy/20 F/4 wk	IMRT	PR
Left sacroiliac joint	40 Gy/20 F/4 wk	IMRT	PR
Lymph nodes of neck, supraclavicular, and superior mediastinal	6 Gy/3 F/3 d	IMRT	SD
Inferior lobe of right lung	30 Gy/3 F/7 d	SBRT	PD
PR: partial response; SD: stable disease; PD: progressive disease; IMRT: intensity-modulated radiation therapy.			

## 2 讨论

NUT癌是近年来提出的一类起源完全独立于人体已有组织细胞的恶性肿瘤，其发病罕见，恶性程度高，预后差^[12]^。其发病机制被认为是*NUT*与*BRD4*形成的融合基因促进了组蛋白乙酰化，从而激活多条生长信号通路，进而形成肿瘤^[1]^。Giridhar等^[3]^回顾分析了2017年来所报道的119例NUT癌病例，发现其好发部位为头颈、肺及纵隔，其中位生存期仅5个月。NUT癌病组织来源不明，有研究^[13-15]^认为NUT癌的细胞形态更倾向于鳞癌，而也有报道认为更倾向于未分化癌，因此对NUT癌的病理诊断尤为困难，免疫组化或FISH检测到NUT-BRD4融合蛋白或*NUT*异位基因是目前诊断的金标准。本病例在外院行手术切除后病理初步诊断为肺小细胞癌，因肺NUT癌病理倾向于低分化或未分化癌，且该病罕见，一般医院难以接触到此类病例，故容易造成误诊。后患者于我院进行病理会诊，经FISH检测最终确诊为肺NUT癌。

肺是NUT癌的好发部位之一，但对肺NUT癌的报道较少，最大规模集中报道是2015年发表在*Journal of Thoracic Cancer*上的一项回顾性分析^[8]^，该研究回顾性分析了8例肺NUT癌的临床特征，不幸的是8例患者的平均生存期仅2.2个月，远低于Giridhar等^[3]^报道的119例NUT癌的5.5个月平均生存期。有关肺NUT癌的个案报道^[9-11, 16-18]^均表明肺NUT癌治疗困难，预后不佳（表 2）。本病例经过手术、化疗、抗血管治疗、放疗等综合治疗，其预后明显好于大多数有关肺NUT癌的报道，从诊断该疾病到现在已生存超过24个月，目前总生存期（overall survival, OS）仍未达到。

**表 2:** 总结近年来有关肺NUT癌的病例报道 Clinical features of patients diagnosed with pulmonary NUT carcinoma in recent years

References	Age (yr)	Gender	Symptoms	Treatment	OS (mon)
Karakus, *et al* (2017)^[4]^	6	NA	NA	NA	NA
Sholl, *et al* (2015)^[8]^	21-68 (8 cases)	F/M	Cough	Chemotherapy and radiation and resection	Median 2.2
Cao, *et al* (2017)^[9]^	48	M	Bloody sputum and shortness of breath	Resection	6
Parikh, *et al* (2013)^[10]^	36	M	Cough and chest pain	Chemotherapy and radiation and clinical trial	3
Harms, *et al* (2015)^[11]^	31	F	NA	NA	NA
Tanaka, *et al* (2012)^[16]^	7 and 14	F and M	Cough and chest pain	Chemotherapy and radiation	4 and 12
Maur, *et al* (2015)^[17]^	21	NA	Fatigue, fever, chest pain	Chemotherapy and radiation	NA
Benito Bernaldez, *et al* (2016)^[18]^	23	M	Chest pain and asthenia	Chemotherapy and BET inhibitor	3
Chai, *et al* (2020)^[23]^	26 and 69	M and M	Shortness of breath and cough	Chemotherapy	5 and 6
F: Female; M: Male; NA: not available.					

NUT癌之所以预后差与其快速复发，进展速度快，治疗效果欠佳相关。在大多数肺NUT癌的病例报道中患者即便是初诊为早期，及时进行了手术，仍然在短时间内复发，快速进展，OS极短^[9, 16]^。而初诊即为全身多处转移的患者预后更差^[17]^。本病例术后辅助治疗后无进展生存期（progression-free survival, PFS）达12个月，这提示局部治疗后加强全身治疗，消除亚临床病灶或许可延长复发时间。但自从患者纵隔复发后肿瘤便此起彼伏，且生长速度快，肿瘤负荷大。目前为止，对于NUT癌没有标准的治疗推荐^[19]^。Giridhar等^[3]^回顾性分析了119例NUT癌的治疗预后关系，发现放疗是影响预后的一项独立正向因素，超过50 Gy或许更能获益。本病例中第一次术后复发放疗计划先行IMRT 40 Gy/20 F放疗，后局部缩野加量，但第一阶段放疗完成后患者因个人原因未行加量照射。后续多次复查影像学提示经40 Gy照射的部位并未再复发。但后续肋骨微小转移灶未经放疗2个月后肿瘤明显增大，提示NUT癌生长速度迅速，倍增时间短，可能对放疗高度敏感。综合患者高龄、基础疾病多，后续膈肌、髂骨转移灶放疗均给IMRT 40 Gy/20 F照射，放疗后肿瘤均消退明显。甚至后续锁骨上、纵隔淋巴结低剂量放疗IMRT 6 Gy/3 F也能使肿瘤稳定。但后续肺内转移灶行SBRT 30 Gy/3 F并未使肿瘤再次得到缓解。本病例提示调强放疗对肺NUT癌敏感，但立体定向放疗却不敏感，这值得进一步研究。回顾病例，仔细观察可以发现患者后续锁骨上及纵隔转移淋巴结在膈肌放疗后复查时即有显影，提示我们定义靶区时应尽可能包括所有的可疑转移灶。化疗在NUT癌的治疗中没有标准，有几例个案提到针对肉瘤的化疗药物如长春新碱、环磷酰胺、阿霉素类可能使NUT癌获益^[2, 20-22]^。本病例化疗方案的选择为依托泊苷和顺铂以及卡铂，主要考虑其病理特征以及睾丸蛋白基因参与发病是否与生殖来源肿瘤相关，生长迅速，肿瘤负荷大，可能对生殖系肿瘤化疗方案敏感。但因其作为术后辅助化疗，且复发后主要以放疗为主，并不能提示NUT癌对该化疗方案是否敏感。此外，本病例考虑到NUT癌生长迅速，遂选择了抗血管药贝伐珠单抗以及后续的安罗替尼。Chai等^[23]^报道了1例鼻腔NUT癌采用放疗联合安罗替尼治疗延长了患者生存期。随着近年来对NUT癌的发病机制的深入研究，关于NUT癌的靶向药正处于如火如荼的研发中。其中已有部分药物进入临床试验，初步展现出疗效^[13, 24]^。本病例后续的免疫治疗以及安罗替尼均属于探索性治疗，免疫治疗并没有展现出让人惊喜的疗效，而安罗替尼的疗效有待后续观察。

**图 7:**
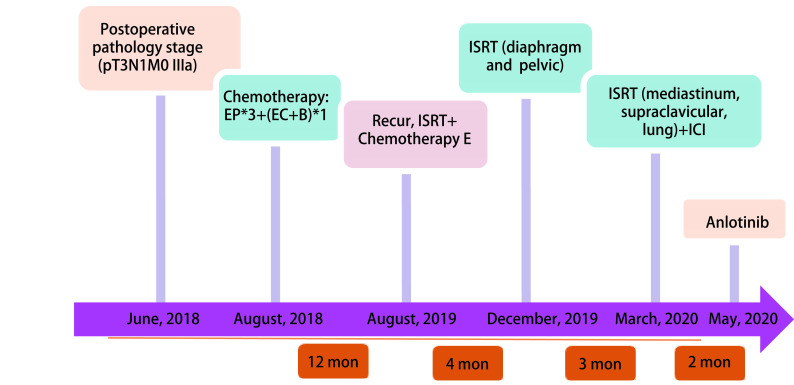
诊疗经过概述 Overview of the whole treatment process. EP: VP-16+DDP; EC: epirubicin+cyclophosphamide.

总之，该病例的报道帮助我们对肺NUT癌有了进一步的认识，肺NUT癌经过手术，适当的化疗、抗血管治疗以及放疗等综合治疗后获得较长的生存期。本病例向大家展示了肺NUT癌对放疗非常敏感，且达到一定剂量照射后不易复发。但亚临床病灶可迅速复发转移，生长速度极快，术后应加强全身治疗，加强监测力度，推荐最长2个月复查一次，有症状者应及时复查，尽早发现微小转移灶，放疗靶区尽可能包括所有微小病灶，剂量可据情况而定，放疗技术不推荐立体定向体部放疗（stereotactic body radiation therapy, SBRT）。肺NUT癌强调全程管理，初治尽可能的将肿瘤负荷降到最低，后续加强监测，及时发现复发转移灶，尽快放疗，或许是延长生存期的关键。
